# Cognitive Functioning in Older People. Results of the First Wave of Cognition of Older People, Education, Recreational Activities, Nutrition, Comorbidities, Functional Capacity Studies (COPERNICUS)

**DOI:** 10.3389/fnagi.2018.00421

**Published:** 2018-12-21

**Authors:** Sławomir Kujawski, Agnieszka Kujawska, Małgorzata Gajos, Weronika Topka, Radosław Perkowski, Joanna Androsiuk-Perkowska, Julia L. Newton, Paweł Zalewski, Kornelia Kędziora-Kornatowska

**Affiliations:** ^1^Division of Ergonomics and Exercise Physiology, Department of Hygiene, Epidemiology and Ergonomics, Collegium Medicum in Bydgoszcz, Nicolaus Copernicus University in Toruń, Toruń, Poland; ^2^Department and Clinic of Geriatrics, Collegium Medicum in Bydgoszcz, Nicolaus Copernicus University in Toruń, Toruń, Poland; ^3^Department of Physiology, Collegium Medicum in Bydgoszcz, Nicolaus Copernicus University in Toruń, Toruń, Poland; ^4^Institute for Cellular Medicine, The Medical School, Newcastle University, Newcastle upon Tyne, United Kingdom

**Keywords:** MMSE, MoCA, TMT B, occupational status, education, aerobic capacity

## Abstract

**Background:** Cognitive reserve is a way of explaining why some individuals with a high degree of brain pathology are without clinical manifestations. In this study, factors related to systemic diseases, body composition, aerobic capacity, past and current behavior were examined and included as predictors of cognitive function.

**Materials and Methods:** 407 subjects (60–88 years old) underwent physical examination and cognitive function assessment [Mini-Mental State Examination (MMSE), Montreal Cognitive Assessment (MoCA), and Trail Making Test Part B (TMT B)]. Predictors of cognitive functioning were evaluated: occupational status (OS), diet, mental and touristic activities were assessed using an ad hoc questionnaire. Aerobic capacity was measured using a six-minute walk test (6MWT).

**Results:** With each year of age there is a decrease in MMSE score by 0.18 points. Varicose veins on lower extremities and low OS were also significantly associated with MMSE result. For every year of having hypertension, low OS and not being abroad in the last 3 years, there was 0.17, 0.30, and 0.16 less points, respectively, and 0.15 more point per one additional meter walked in 6MWT in the MoCA score. With each year of age and for low OS there was there were 0.31 and 0.21 s more to complete TMT B, respectively.

**Conclusion:** Education, OS, presence of systemic diseases and social and tourist activities, aerobic capacity and body composition could be considered as factors contributing to cognitive functioning in older people. However, the relationship of above mentioned factors with education level and cognitive function may be not fully orthogonal.

## Introduction

Aging is inevitable for all living creatures, however, it seems that an individual’s behavior and interaction with the ambient environment could mitigate the rate at which an individual moves toward the point of performance disturbance. The concept of “cognitive reserve” promoted by Stern (2002) assumes that one can increase resistance of cognitive processes to brain damage. Over decades research has shown modifiable factors such as education (Katzman, 1993), occupation (Stern et al., 1994; Maguire et al., 2000), environment and physical activity (Brown et al., 2003), mental activities such as reading (Wilson et al., 2000) and bilingualism (Craik et al., 2010) and its influence on deceleration of cognitive decline dynamics or postpone the time point of cognitive decline during aging.

What makes a cognitive reserve modelcomplex is the fact that educated people are more often high-occupation workers, and in turn have higher socioeconomic status (SES): education, income, and occupation contribute to decrease of risk factors for cardiovascular disease (Winkleby et al., 1992) which, on the other hand, could be related to cognitive impairment in older people (Breteler et al., 1994). Moreover, a higher level of education is related to undertaking a pro-health behaviors such as abstaining from smoking (Stern et al., 1994). Conversely, Poblador-Plou et al. (2014) showed that hypertension and diabetes are two of the most frequent comorbidities with dementia; results of statistical analysis revealed that the comorbidities significantly related to dementia were, inter alia, congestive heart failure, cerebrovascular disease, cardiac arrhythmia, osteoporosis, thyroid disease, retinal disorders, and anxiety and neurosis (Poblador-Plou et al., 2014). Therefore, one can suppose that higher levels of education could promote cognitive reserve by indirect pathways such as increased awareness or/and funds for personal health care, pro-health behaviors, occupation attainment, more funds on physical, mental and social activities which could promote individual health, what in turn would be related to less rapid cognitive decline. Moreover, it seems that there is close interaction with biological factors, composing brain reserve, with cognitive reserve. It is observed in inter alia studies on bilingualism (Perani et al., 2017). Interrelationships between all of the above variables with aging should be specified. It was shown that cognitive reserve fully mediates the correlation between inter alia education level and cognitive functioning, and age exerts a direct effect on cognitive functioning (Caffò et al., 2016). In addition, authors of a recent study determined a task-invariant cognitive reserve network which presumably supports the concept on differences in brain processing due to the life experiences that might provide reserve on a functional level (Stern et al., 2018). Moreover, results of longitudinal studies on healthy adults indicated that higher memory functioning at the baseline was related to a lesser extent of shrinkage of the lateral prefrontal cortices (Persson et al., 2016). The results mentioned above could serve as evidence against unidirectional interaction in the brain-cognition relationship, therefore we would refer to the cognitive reserve only.

Interestingly, there are cognitive reserve components that are relatively easy to modify across the lifespan, what in turn could lead to successful aging (Lara et al., 2017). Indeed, a long history of researches on influence of physical exercise on cognitive function in older population should be underlined (Kramer et al., 2006). However, physical activity is a very general definition, therefore it should be subdivided due to different pro-health benefits of various energy expenditure patterns (Melanson, 2017). In addition, a more indirect long-term influence of regular physical activity on cognitive functioning decline in older age could be explained in terms of reducing risk and alleviating the symptoms of systemic diseases such as diabetes, hypertension or chronic liver diseases, which in turn can lead to increased rate of cognitive decline during lifetime (Feinkohl et al., 2014; Abraham et al., 2016; Seo et al., 2016).

COPERNICUS study aims to examine the influence of lifestyle and systemic health factors on cognitive decline in a longitudinal manner based on large cohort of older people. The relationship between selected aspects of the participants current and past behavior and health state with cognitive functions analyzed based on data from the first wave of the study is presented in the above studies.

## Materials and Methods

### Study Group

Participants were enrolled into studies based on advertisement using regional TV and radio, during health-promoting lectures, in Day Care Centers for the Elderly, and at various meeting-groups for older people. Message included information on an opportunity to take free-of-charge physical, physiotherapeutic, dietary, social and cognitive assessment for people 60 years old and over. To collect the most representative sample of older cohort as possible, lack of any other excluding factors was underlined. Examination was conducted in the Department and Clinic of Geriatrics, Collegium Medicum University Hospital in Bydgoszcz, Poland. A total number of 407 (aged 60–88 years old) subjects were examined. The study was approved by the Ethics Committee, Ludwik Rydygier Memorial Collegium Medicum in Bydgoszcz, Nicolaus Copernicus University, Torun. Written, informed consent was obtained from all participants.

### Assessment Methods

#### Cognitive Tests

Neuropsychological tests were conducted by the two staff, who underwent common training in the procedures. Almost all (94.2%) neuropsychological tests were conducted by the same person (SK). First, a questionnaire on subjective memory complaints was conducted. Cognitive functioning was assessed with Mini-Mental State Examination (MMSE), Montreal Cognitive Assessment (MoCA), and Trail Making Test Part B (TMT B).

Mini-Mental State Examination is a well-known 30-points questionnaire used in neuropsychological assessment; it measures orientation to time and place, immediate recall and short-term verbal memory, calculation, language, and construct ability (Folstein et al., 1983). Higher score indicates better cognitive performance.

The MoCA assesses several cognitive domains (Nasreddine et al., 2005). It measures all main cognitive domains; namely Visuospatial skills, short-term memory recall, executive functioning (examined by a mini-form of TMT B, phonemic fluency task and a two-item verbal abstraction task). Attention, concentration, and working memory as well as naming and other language skills are evaluated. In the MoCA test, the result of two subtests (Verbal Fluency subtest and Delayed Recall of five nouns) were taken into account separately during analysis. Verbal Fluency subtest result is the number of words in Polish starting with letter “S” which are not own nouns (conjugation prohibited). In the case of Delayed Recall, two scores were taken into analysis: first was the number of words recalled without the help of person carrying out the test. The second score was the overall number of words recalled without help and number of words recalled after category of the word recalled and number of words correctly chosen from list of three words. 24 as an optimal cut-off score in differentiating patients with Mid Cognitive Impairment was chosen based on previous researches on adaptation of MoCA in the Polish sample (Reitan, 1958).

Trail Making Test Part B is a fast-to-assess neuropsychological tool, which measures various skill from executive functioning domain: visuospatial skills, task switching and working memory to mention a few (Magierska et al., 2012).

### Aerobic Capacity Measurement

A six-minute walk test (6MWT) was performed (ATS statement: guidelines for the six-minute walk test, 2002). The testing area was indoor and a flat corridor with distance of 50 meters. To reduce time spent turning, no practice walking was performed before the actual test started. Participants were asked to walk as fast as they were able, and to maintain the same velocity during the whole test. Moreover, participants were reminded twice about the duration of the test and to maintain the same velocity through the whole test. Most subjects walked alone, if not, sufficient interval between consecutive participants was provided to exclude competitive conditions (Roomi et al., 1996).

### Body Composition Analysis

Weight was measured using Tanita BC-545 body-fat analyzer. The measuring method used by the analyzer is bioelectric impedance analysis (BIA) made through ball-of-foot and hand electrodes. Weighing accuracy is 0.1% (Nuñez et al., 1994). Participants were weighed in light clothing. Respondents themselves gave information about height and then BMI was calculated in accordance to WHO recommendations.

### Social

#### Questionnaire

Subjects were asked about overall years spent on education and education level coded as: primary education, vocational school education, secondary education (incomplete), secondary education, higher professional/engineering education, master’s degree, Ph.D. degree, and higher. Occupational status (OS) was coded as: white collar worker, white collar worker in a managerial position, owner of the craft/entrepreneur, military/policeman/other uniformed services, seller/employee of trade, farmer in an individual farm, physical worker-qualified, unskilled worker; the last three was then recoded as “low occupational status” and the rest as “high occupational status.” While eventually creating a binary variable, the highest OS during lifetime was taken into account.

Information collected regarding the last year included questions on the following mental activities: reading press, reading books, watching TV, listening to the radio, going to the café, restaurant, going to the cinema, going to the theater, to the concert, going to church, going to visit friends or family, taking part in social group meetings, computer use, card game, chess/checkers, solving crosswords. Answers on questions about frequency of current mental activities were coded in the following way: “never” was coded as 0, “once a year” as 1, “several times a year” as 2, “1–2 times a month” as 3, “once a week” as 4, “few times a week” as 5, “daily” as 6.

Traveling abroad in the last 3 years was coded in the following binary way: “no” as “0” and “yes” as “1.”

### Physical Examination

All of the examinations were done in the doctor’s office, while the physician was mostly casually dressed to minimize the possible influence of the ‘white-coat’ effect. Blood pressure: Systolic Blood Pressure (sBP) and Diastolic Blood Pressure (dBP) were measured on one upper limb followed by another. Every examination was taken after at least 5 min of sitting in an upright position. The mean value for each sBP and dBP from these two measurements were analyzed.

Pulse pressure (PP) was calculated using formula:

PP=sBP−dBP

Physical and subjective examination was collected during the same visit focusing on history of systemic diseases. Questions were asked on, inter alia, presence of varicose veins on lower limbs, presence of hypertension, duration of hypertension, frequency of blood pressure measurement and presence of diabetes. The presence of any feature were coded as “0” (no) or “1” (yes). Duration of hypertension was expressed in years (numerical variable).

### Statistical Analysis

Mean values and standard deviation of mean are provided in tables. Analysis of differences between male vs. females subgroups was made using *t*-test. General linear regression model was used to predict cognitive function test results. Values of categorical predictors included into models encoded as dummy variables “0” or “1.” Minimum level of tolerance value of predictors included into models was set on 0.20 (Menard, 1995). Adjusted R2 values were used to compare models in their explanation potential of observed variance in cognitive test results. Given R2 values are adjusted one, according to formula:

$$R2adjusted=1-\left[\frac{\frac{\text{SS Residual}}{df}}{\frac{\text{SS Total}}{df}}\right]$$

All statistical analyses were performed using statistical package STATISTICA 13.1 (StatSoft, Inc.).

## Results

The mean age of the examined sample was 69.8 years with a range from 60 to 88 years old. The MMSE score was 27.6 points, while MoCA was 23.2 points. In the Delayed Recall subtest of MoCA test subjects recalled 2.3 on 5 words without the help of a test administrator, and 4.7 on 5 with help. In the Verbal Fluency subtest of MoCA subjects pronounced 12.37 words per minute (this was the mean). 149.5 s were needed to complete TMT B. Participants spent a mean of 13.96 years on education. Mean systolic blood pressure during supine was above 140 mmHg. Descriptive statistics are provided in the Table 1.

**Table 1:** Descriptive statistics.

Variable	Mean	Minimum	Maximum	*SD*
Age	69.76	60	88	6.14
Age of memory complaints	64.97	40	83	7.49
MMSE	27.56	16	30	2.26
MoCA	23.21	9	30	3.67
MoCA DR	2.27	0	5	1.62
DR in overall	4.7	1	5	0.67
Verbal fluency	12.37	0	24	4.49
TMT B	149.46	43	628	95.02
Length of hypertension history	14.09	0.5	60	10.98
sBP supine	140.35	77	200	18.59
dBP supine	85.06	55	120	10.59
mBP supine	120.04	59.4	164.2	15.54
PP supine	55.28	-12	120	14.37
Years of education	13.96	7	26	3.31
6MWT	494,00	100	975	101.54
Body fat [%]	33.71	8.4	52.7	8.29
Mental activities	41.39	18	60	8.43


*MMSE, Mini-Mental State Examination; MoCA, Montreal Cognitive Assessment; DR, Delayed Recall subtest of MoCA; TMT B, Trail Making Test Part B; sBP, systolic blood pressure; dBP, diastolic blood pressure; mBP, mean blood pressure; PP, pulse pressure; 6MWT, six-minute walk test.*

As Table 2 shows, there was a 407 participants examined (95 males). Interestingly, 76.2% of participants declared memory complaints, while assuming < 24 cut-off point in MoCA scale about a half could be diagnosed as having mild cognitive impairment.

**Table 2:** Demographic description of sample.

Variable	No.	%
**Sex**		
M	95	23.34
F	312	76.66
**Subjective memory complaints**		
No	97	23.83
Yes	310	76.17
**Objective cognitive impairment MoCA < 24**		
No	203	49.88
Yes	204	50.12


A statistically significantly worse performance of Delayed Recall subtest of MoCA was observed in males (1.82 vs. 2.4 words recalled in females, *p* = 0.003). Moreover, males were characterized by higher dBP values (87.58 vs. 84.3, *p* = 0.015), % of body fat (36.24 vs. 25.73, *p* < 0.00001), less frequent mental activity (*p* = 0.004).

Two hundred and thirty three subjects had diagnosis of hypertension, 63 were diagnosed with diabetes. During physical examination, varicose veins on lower extremities were observed in 26.5% of the sample. Moreover, 20.6% participants had education lower than a complete secondary education. 16.2% of subjects were low-skilled employees (unskilled and physical workers), whereas 80.8% were highly-skilled employees (white-collar workers or/and owners of crafts, managers).

General regression model includes the following explanatory variables: age, duration of hypertension, years of education; all three variables are expressed in years. Moreover results of 6MWT test (expressed in meters walked during of the test), % of body fat and overall mental activity rate, supine PP were added as continuous predictors of cognitive tests results. Sex, diabetes, varicose veins on lower limbs, being hired as low employee/high employee, traveling abroad in the last 3 years were added as discrete predictors. Selected discrete variables are presented in the Table 3.

**Table 3:** Discrete descriptive statistics.

	No of	% of
Hypertension based on history	participants	sample	MMSE	MoCA	DR	DR in overall	VF	TMT B
No	174	42.75	27.72 (2.1)	23.53 (3.5)	2.35 (1.6)	4.69 (0.6)	12.57 (4.5)	140.94 (92.9)
Yes	233	57.25	27.44 (2.3)	22.97 (3.8)	2.20 (1.7)	4.70 (0.7)	12.22 (4.5)	155.83 (96.3)
**Hypertension based on Our measurement**								
No	218	53.56	27.63 (2.2)	23.41 (3.7)	2.29 (1.7)	4.70 (0.7)	12.76 (4.3)	144.38 (96.1)
Yes	189	46.44	27.48 (2.3)	22.98 (3.6)	2.24 (1.6)	4.69 (0.7)	11.93 (4.6)	155.29 (93.6)
**Diabetes?**								
No	341	83.78	27.60 (2.3)	23.40 (3.6)	2.35 (1.6)	4.72 (0.6)	12.60 (4.5)	145.53 (92.3)
Yes	63	15.48	27.38 (2.1)	22.29 (3.6)	1.83 (1.5)	4.57 (0.9)	11.37 (4.5)	162.19 (99.8)
Lack of data	3	0.74						
**Varicose veins on lower limbs**								
No	299	73.46	27.75 (2.1)	23.40 (3.6)	2.29 (1.7)	4.68 (0.7)	12.47 (4.6)	143.63 (93.4)
Yes	108	26.54	27.06 (2.5)	22.68 (3.8)	2.19 (1.5)	4.74 (0.7)	12.09 (4.1)	165.62 (98.0)
**Low/high occupational status**								
Low	66	16.22	26.30 (2.6)	20.89 (3.4)	1.48 (1.4)	4.47 (0.9)	9.50 (3.8)	207.64 (109.1)
High	329	80.84	27.80 (2.1)	23.59 (3.6)	2.40 (1.6)	4.74 (0.6)	12.89 (4.4)	138.93 (88.5)
Lack of data	12	2.95						
**Education level**								
Primary education	9	2.21	25.11 (2.7)	19.56 (4.4)	1.44 (0.9)	4.44 (0.5)	8.00 (4.4)	261.11 (149.7)
Vocational school education	62	15.23	26.37 (2.8)	21.27 (3.7)	1.68 (1.6)	4.44 (1.0)	9.79 (3.9)	210.82 (119.2)
Secondary education (incomplete)	13	3.19	27.38 (2.4)	20.69 (3.4)	1.69 (1.4)	4.92 (0.3)	9.08 (3.9)	148.92 (53.7)
Secondary education	183	44.96	27.72 (2.0)	23.51 (3.6)	2.52 (1.6)	4.77 (0.6)	12.57 (4.3)	144.36 (88)
Higher professional/engineering education	53	13.02	28.11 (2.3)	23.72 (3.4)	2.09 (1.6)	4.74 (0.7)	13.49 (4.1)	126.87 (61.4)
Master’s degree	80	19.66	28.03 (1.8)	24.28 (3.1)	2.41 (1.7)	4.68 (0.6)	14.11 (4.4)	120.42 (80)
Ph.D. degree and higher	2	0.49	28.00 (1.4)	26.00 (2.8)	2.00 (2.8)	5.00 (0.0)	15.50 (2.1)	97 (0)
Lack of data	5	1.23						
**Did travel abroad as in the last 3 years**								
No	186	45.70	26.76 (2.7)	22.39 (3.9)	2.15 (1.6)	4.67 (0.7)	12.03 (4.5)	160.17 (99.8)
Yes	221	54.30	27.4 (2.4)	23.6 (3.4)	2.4 (1.6)	4.71 (0.7)	12.64 (4.5)	139.14 (89)


The same general linear model with above mentioned predictors included was used to predict all of cognitive tests results. It explained 20% variance in MMSE. With each year of age more, there is a decrease in MMSE score by 0.18 points. For not having varicose veins on lower extremities there is 0.17 increase and for low occupational status there is a decrease of -0.23 points in MMSE, respectively. In the case of MoCA, model explained 30% of variance. Significant predictors were: duration of hypertension, 6MWT result, low occupational status and not traveling abroad in the last 3 years. For every year of having hypertension there is 0.17 less points in the MoCA score. Moreover, for additional meters walked in 6MWT there are 0.15 more points in the MoCA score. For low occupational status and not being abroad in the last 3 years, there was 0.30 and 0.16 less points in the MoCA score. In the case of words recalled without help of test administrator in DR subtest of MoCA 8% of variance was explained. For low OS there was 0.24 less word recalled. Moreover, in the case of overall score of DR, model explained 9% of variance. For every meter walked more in 6MWT there was 0.17 more word recalled, while for having low OS there was 0.32 less words recalled. Moreover, in the Verbal Fluency subtests of MoCA for low OS there were 0.21 less words recalled and the model explained 18% of variance in results of this subtest. In the case of TMT B result there was two significant predictors: for every year of age more there was 0.31 and for low occupational status there was 0.21 s more to finish this test and the model explained 28% of variance in TMT B results.

## Discussion

In conclusion, age, duration of hypertension, years of education, the highest occupational status obtained during alifetime, aerobic capacity, % of body fat and overall mental activity rate, pulse pressure, sex, presence of diabetes, varicose veins on lower limbs, and traveling abroad in the last 3 years could be considered as factors contributing to level of cognitive functioning in older people. The model of general regression analysis applied in analysis of data obtained from a large cohort study of older people comprehensively studied explained the biggest part of variance in the case of the TMT B and MoCA overall score, while the smaller part was explained in the case of MMSE and MoCA subtests. It is not surprising, as MoCA, in comparison to MMSE is more effective tool in detection of cognitive decline in aging (Gluhm et al., 2013) which potentially could explain observed differences. The TMT B test has high ecological value, confirmed by, inter alia, its acceptable accuracy as a predictor of older driver on-road performance (Classen et al., 2013) or as a factors related to falls in older population (Caetano et al., 2018).

Values of most of the variables included into our statistical model as predictors might have a direct as well as indirect relationship with cognitive function. Results of previous studies showed the relationship between cognitive decline and pulse pressure (Obisesan et al., 2008; Waldstein et al., 2008). Higher PP could be a surrogate marker of increased arterial stiffness (Laurent et al., 2006), which could indirectly be related to cognitive function through increasing various cardiovascular and metabolic risk factors and inflammation markers (Mattace-Raso et al., 2004; Scuteri et al., 2004). Moreover, a direct mechanism of increased arterial stiffness influence on cognitive function could be also be taken into account. The brains lack of protection from the elevated pulsatilty of blood flow, could lead to an injury of distal vessels in the brain. Moreover, increased PP may lead to micro- and macro-vascular disease, disturbed cerebral perfusion and integrity of the blood–brain barrier (O’Rourke and Safar, 2005). In addition, varicose veins occurrence might be related to peripheral artery disease, although some confounding factors may be involved (Chang et al., 2018). In turn, results of epidemiological studies showed that an indicator of peripheral arterial disease was associated with an increased risk of Vascular Dementia (Newman et al., 2005; Laurin et al., 2007). In addition, our results showed that aerobic fitness significantly predicted results of MoCA and its subtest Delayed Recall, in turn, arterial stiffness are significantly lower in individuals with a higher aerobic capacity (Santos-Parker et al., 2014). Interestingly, many questions on effects of fitness level on cognitive function remains unanswered (Kramer and Colcombe, 2018).

Body fat level also could contribute to cognitive function in direct as well, in an indirect way. Obesity was thought as one of risk factors of cognitive disturbances in cardiac patients (Alosco et al., 2014). Adipokines, hormones produced by fat tissue, could serve as a link helpful in explanation of co-occurrence of increased body fat level with risk of dementia (Kiliaan et al., 2014). Moreover it might play a role in an indirect way by, inter alia, increasing insulin resistance. In turn, type 2 diabetes mellitus is related with both insulin resistance and cognitive dysfunction and increased body fat level (Biessels and Reagan, 2015).

Traveling abroad in the last 3 years which was positively associated with MoCA score, is a type of activity which has several subcomponents which could potentially enhance cognitive reserve; increased level of physical activity, increased mental stress by undertaking navigation and communication tasks, multi-senses stimulation, to name a few. In turn, mental activities could slower the rate of cognitive decline in demented patients (Cheng et al., 2014) and positive relationship between overall mental activities and cognitive tests results was observed in the above studies also. Moreover, its seems reasonable that being able to travel abroad could be an indirect indicator of positive subjective assessment of own physical and mental capabilities and high financial status, which the latter in turn is related to slower cognitive decline in elderly (Lyu and Burr, 2016). Moreover, observed sex differences in favor for women are in line with previous studies (Jagger et al., 2016; McCarrey et al., 2016) what could be explained by differences in sex hormones pool, especially in the mid-life or indirectly, by gender differences in pattern of social activities (Su et al., 2009).

It is noteworthy that models enriched by questions on current and past regular physical exercise, current diet or tourism activities did not increase the rate of explained variance in results of cognitive tests results. These types of behaviors could contribute to increased cognitive reserve indirectly by improving overall health state; decreasing the risk of systemic diseases, allowing a more cognitive-stimulating lifestyle, better body composition and higher aerobic capacity therefore adding them into the model did not increase its explanatory potential.

A Brazilian study showed that a subgroup of patients with high educations (≥9 years) performed better in TMT B in than those subgroups with less education (Hamdan and Hamdan, 2009). This was shown in the normalization study of TMT also (Perianez et al., 2007). Year of education applies to the first 2 or 3 decades of life, therefore if it would be the most important explanatory variable of cognitive function in older people, thus public health guidelines should be revised. Based on that, one can suppose a critical period beyond which undertaking activities oriented on increasing cognitive reserve would be unsuccessful. On the other hand, our results showed that low occupational status (unskilled or physical worker) is a significant predictor of all used cognitive function tests in the above researches. Interestingly, in comparison to years spent on education, occupational status applies further than the first decades of life, although is related to education level, therefore these predictors may be not fully orthogonal (Opdebeeck et al., 2016). Therefore, predictors of cognitive functioning used in the above study shows an explanatory potential in older people in factors related to life-span pattern of behavior. Moreover, based on the above results, it should be highlighted that, beyond the classical three dimensions of cognitive reserve (education, occupation, and leisure activities), a number of other clinical factors, such as supine pulse pressure, duration of hypertension, % of body fat, presence of diabetes and varicose veins on lower limbs presumably could also be added as another dimension of cognitive reserve. Above results were obtained in Polish sample, therefore it would be worth to conduct studies with same methodology in other countries and compare results. Further studies in longitudinal manner are needed to estimate the influence of undertaking particular behaviors throughout lifetime on cognitive reserve development. Moreover, it is worth examining if there are cognitive tests which results are associated with cognitive reserve minimally or not at all (Lavrencic et al., 2018). Most importantly, some variables of cognitive reserve are modifiable, therefore it was proposed that interventions during the lifetime that aim to support independent functioning and improving quality of life may lead eventually to successful aging (Lara et al., 2017).

## Conclusion

Age, sex, indicators of systemic diseases, arterial stiffness, aerobic capacity and body fat percentage, years of education, frequency of current overall mental activities, traveling abroad in the last 3 years, and the highest occupational status obtained during lifetime combined together created a set of variables significantly predicting current cognitive function in a population of older people. Based on these results, it is worth considering factors beyond that of education level such as: occupational status, presence of systemic diseases, mental and tourist activities, aerobic capacity and body composition as factors contributing to cognitive functioning in older people. However, the relationship between the mentioned factors with education level and cognitive function may be not fully orthogonal, therefore further studies on cognitive reserve in a longitudinal manner are advised.

## Author Contributions

SK and AK: conceptualization, data curation, formal analysis investigation, methodology, project administration, resources software, supervision validation, writing – original draft, and writing – review and editing. MG, WT, RP, and JA-P: conceptualization, data curation, formal analysis, investigation, methodology, project administration, supervision, writing – original draft, and writing – review and editing. JN: methodology, supervision, and writing – review and editing. PZ and KK-K: methodology, project administration, supervision, writing – original draft, and writing – review and editing.

## Conflict of Interest Statement

The authors declare that the research was conducted in the absence of any commercial or financial relationships that could be construed as a potential conflict of interest.
